# Multiple abdominopelvic abscesses caused by fishbone: A case report of rare etiology and literature review

**DOI:** 10.1016/j.ijscr.2023.108608

**Published:** 2023-08-06

**Authors:** Athary Saleem, Saqer Alenezi, Seddeqah Abdulbaqi, Anas Saud, Nimer Al-Shadidi

**Affiliations:** Department of General Surgery, Al-Adan Hospital, Kuwait

**Keywords:** Abdominopelvic abscess, Perforation, Foreign body, Laparotomy, Fish bone, Case report

## Abstract

**Introduction and importance:**

Foreign body ingestion, particularly fishbone, is a prevalent medical complaint in the emergency department. Usually, these foreign substances pass through the gastrointestinal tract without causing any complications. The clinical manifestations of foreign body consumption are non-specific.

**Case presentation:**

A 32-year-old male patient presented to our hospital with severe abdominal pain. Physical examination revealed a distended abdomen and tenderness. Plain chest and abdominal X-rays were unremarkable. The performed computed tomography (CT) of the abdominopelvic region showed multiple abscesses. Then, an exploratory laparotomy was decided during which a foreign body, a fishbone, was detected and the affected omental mass was resected, and abscess drainage was done. The resected specimen was sent for histopathological studies. The postoperative period was uneventful.

**Clinical discussion:**

Perforation of the intestinal wall by fishbone ingestion is an unusual entity. The clinical features of intestinal perforation are usually non-specific resulting in delayed diagnosis. Based on individual situations, the treatment strategy can be surgical or non-surgical.

**Conclusion:**

Even though ingesting a foreign body is a frequent complaint in clinical practice, its repercussions are extremely rare. Our case presented multiple intra-abdominal abscesses and perforation as a complication of accidental fishbone ingestion.

## Highlights

• Foreign body ingestion consequences, as perforation, are extremely rare.
• The clinical features of intestinal perforation are usually non-specific resulting in delayed diagnosis.
• Computed Tomography is highly sensitive diagnostic tool of intestinal wall perforation.
• The gold standard of treatment is surgical techniques to remove foreign objects.

## 1 Introduction

Accidental ingestion of a foreign body, as a fishbone, is relatively common, accounting for 48–88 % of foreign substance consumption cases [[1], [2], [3]]. Within a week, the majority of foreign objects pass uneventfully through the gastrointestinal tract after being encased in a bolus of food and moved by the intestinal peristaltic contractions [4,5]. The capability of ingested foreign bodies of impacting, penetrating, or perforating the gastrointestinal wall accounts for less than 1 % [5].

The risk of intestinal perforation is low and significantly less to be occurred in the large intestine [6]. If perforation develops, the patient can present either immediately with acute abdomen or later with intra-abdominal collections [7].

It is estimated that 10 to 20 % of patients require endoscopic intervention to remove a foreign body, and surgical intervention is required in less than 1 % of cases [5,8].

Here we report a 32-year-old gentleman who presented with severe abdominal pain found to be due to ingested foreign body, fishbone, resulting in multiple abscess formation and intestinal obstruction. Our work has been reported in line with the SCARE 2020 criteria [9].

## 2 Case presentation

A 32-year-old male patient, with unremarkable medical or surgical history, presented to our hospital with a ten-day history of severe abdominal pain. This pain was diffused on and off in nature and was associated with vomiting and followed by constipation. Physical examination showed a febrile, paled patient with a distended abdomen with localized tenderness in the left paraumbilical region. No evidence of bleeding or masses was detected in the rectal examination.

On admission, the patient was conscious, alert, and oriented to time, place, and person, and Table 1 showed his vital signs.

**Table 1:** patient's vital signs and laboratory investigations on admission.

Patient's vital signs	Hematology		Coagulation	
GCS 15/15	WBC	23.6H	PT	18.3H
BP 144/80 mmHg	Hb	116 L	PT-INR	1.55
HR 100 bpm	Plt	587H	APTT-A	30.7
RR 22 bpm	Neutrophils	21.16H	APTT-A Ratio	1.18
SPO_2_ 99 % on room air	Lymphocytes	1.30		
Body temperature 39 °C	Monocytes	0.97		
	Eosinophils	0.14		
	Basophils	0.02		

**Fig. 1:**
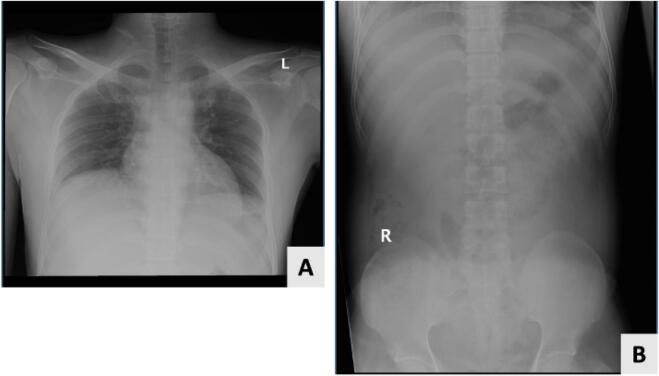
Plain radiographs failed to identify the foreign body. A: Plain chest X-ray. B: Plain abdominal X-ray.

Both performed plain chest and abdominal X-rays failed to diagnose the existence of a foreign body (Fig. 1).

Then, abdominal ultrasound was done before hospital admission, showing two large intraperitoneal abscesses in the right lumbar region with a mild subhepatic thick turbid collection.

**Fig. 2:**
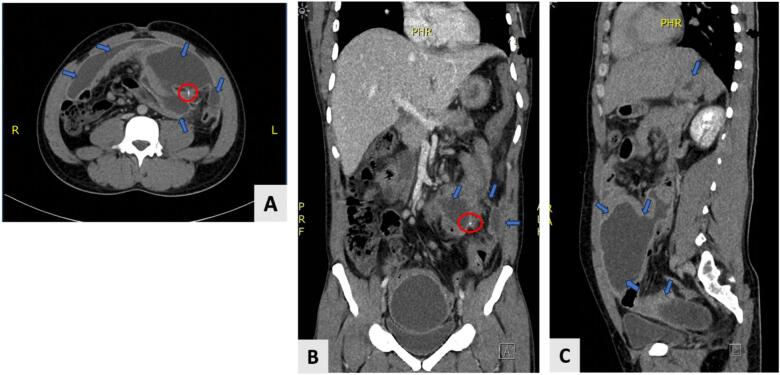
CT scan of the abdominopelvic region. A: Axial view. B: Coronal view. C: Sagittal view. Blue arrows pointing toward multiple sites of abscesses. Red circle encircles a cross section of the foreign body. (For interpretation of the references to colour in this figure legend, the reader is referred to the web version of this article.)

**Fig. 3:**
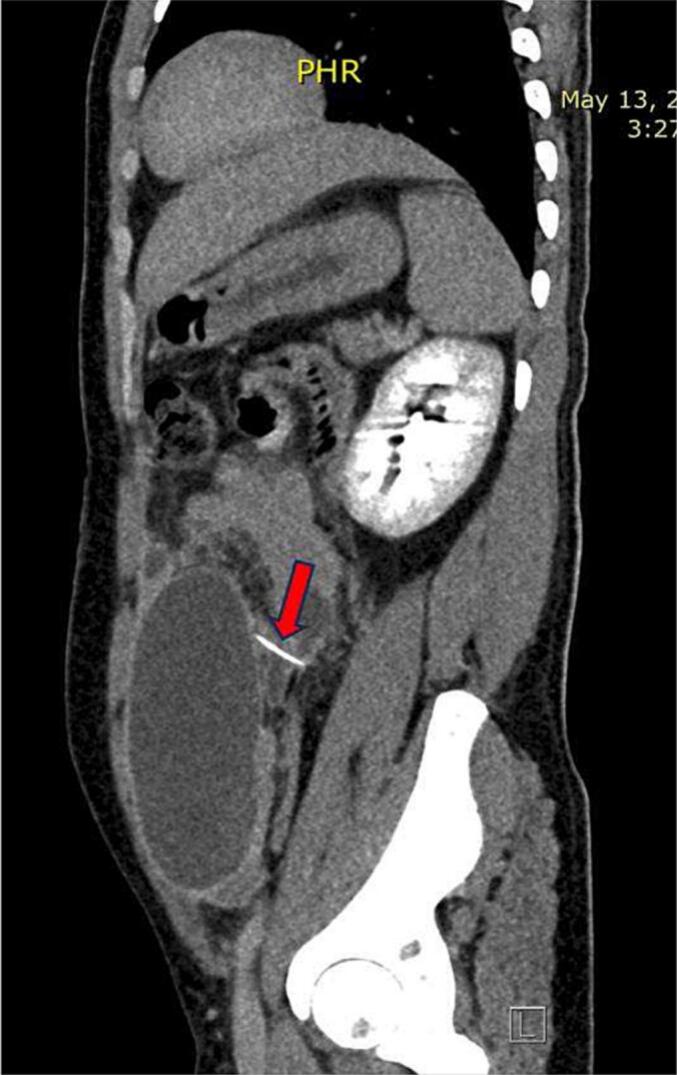
Sagittal view of CT scan of the abdominopelvic region illustrating hyperdense foreign body.

At this point, a computed tomography (CT) scan of the abdominal and pelvic region was decided, revealing (Fig. 2, Fig. 3):

• Multiple encysted fluid collections were seen in the pelvic abdominal region.
• Two encysted fluid collections were seen connecting, one was seen in the anterior part of the right lumbar region measuring about 12 × 3 cm, and another one was seen in the umbilical and left lumbar region measuring about 11.6 × 7 cm.
• Another large, encysted collection was seen in the pelvic region between the urinary bladder and rectum, compressing the urinary bladder anteriorly and measuring about 12 × 7.6 cm.
• Multiple enlarged mesenteric and para-aortic lymph nodes.
• Normal appearance of liver, gallbladder, common bile duct, pancreas, spleen, kidneys, and adrenals.
• Impression was multiple large, encysted collections most likely to be multiple abscesses in the pelvic abdominal region.

**Fig. 4:**
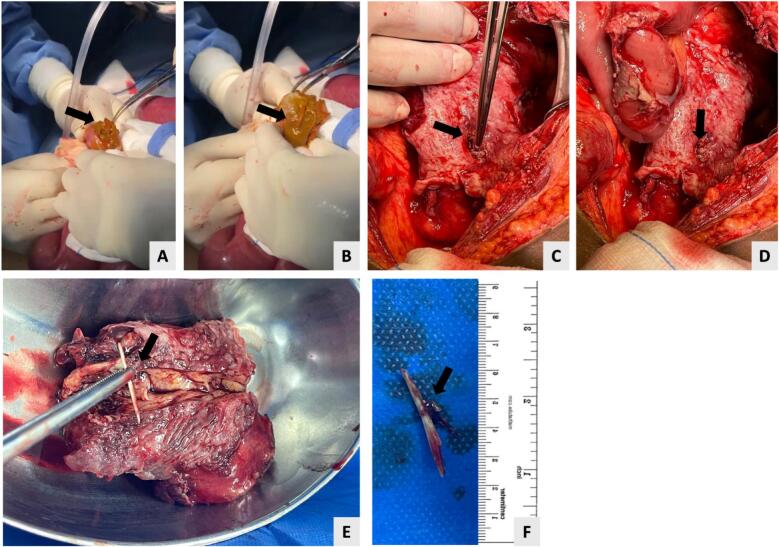
Intraoperative findings. A and B: Intraabdominal abscess drainage. C: Bowel perforated area. D: Focal inflamed area with central necrosis. E: The resected specimen and detected fishbone. F: Foreign body identified as fishbone.

Conservative management was with intravenous fluids and antibiotics initiated, which failed to control the patient's clinical condition. So, a diagnostic laparoscopy was planned (Fig. 4). The procedure was initiated by supra and infra umbilical incisions, during which a large amount of pus spilled through the umbilical trocars and a large space of the abdomen was found to be occupied by pus. Thus, the decision was made to be shifted to the open laparotomy technique. Further adhesions were detected between the small bowel and the pyloric membrane. An omental mass that contains a fishbone, located between the transverse and sigmoid colon, was resected. During mass resection, no perforation was noted, the transverse colon and stomach sides appeared healthy, and the sigmoid colon showed an inflammation reaction. Due to the presence of an inflamed and shrunken omental area located between the transverse colon and sigmoid, the site of perforation was thought to be the colon.

The presence of an inflammatory response indicated healed perforation that was created by the fishbone.

Drainage of supra hepatic abscess and inflamed sigmoid colon was also achieved.

Multiple attempts were done to assess the perforation and abscess formation sites. These include an air leak test, upper gastrointestinal methylene blue test, and milking test. All tests showed negative results. The patient's abdomen was kept open for abdominal re-evaluation, aiming to detect the perforation site.

Three days later, a second look was performed, during which no evidence of free fluid or leak was detected. Running of the small and large bowel was done, and two drains were placed in the left upper quadrant and pelvis. Finally, abdominal skin closure was achieved using skin clips.

**Fig. 5:**
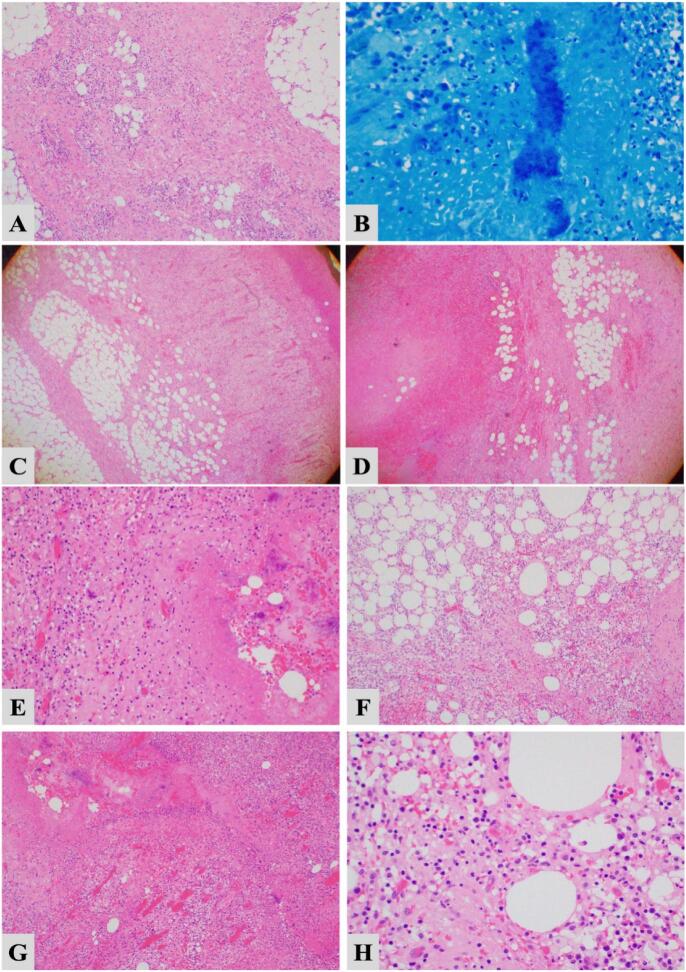
Histopathological study of the resected specimen.

The resected specimen was sent for histopathological studies (Fig. 5).

A: Inflammation and myofibroblastic proliferation.

B: Gram stain inflammation and Bacteria.

C: Inflammation and myofibroblastic proliferation.

D: Necrosis myofibroblastic proliferation.

E: Inflammation giant cell reaction.

F: Inflammation and myofibroblastic proliferation.

G: Inflammation giant cell reaction.

H: Acute inflammation and mesenteric tissue.

Grossly, the resected specimen revealed a tan-brown mass measuring 8.5 × 6.5 × 2.5 cm. The cut sections showed a solid yellow surface with hemorrhagic and necrotic areas. Also, the resected mass was a positive Gram stain for bacterial organisms. So, acute and chronic inflammation with myofibroblastic proliferation, ischemic with necrotic changes, and abscess formation of the omental mass was detected.

The patient could not recall the time of fish bone ingestion retrospectively. The postoperative period was uneventful, and the patient was discharged on postoperative day 5.

## 3 Discussion

The intestinal wall perforation is commonly encountered in long and sharp-edged foreign materials due to the anatomical features and/or restricted angulation of the gastrointestinal tract (GIT). The most prevalent ones are chicken bones, toothpicks, and fish bone, which is the most common etiology of GIT perforation [[10], [11], [12]].

The clinical features of bowel perforation due to fish bone ingestion are variable [13,14]. Abdominal pain, which can be persistent prior to presentation, vomiting, nausea, and fever are common symptoms of patients [10,13,14]. Moreover, the patient's examination can reveal guarding, peritonitis, and rebound tenderness that indicates the perforation site [10,14].

In our case, the patient presented with severe abdominal pain and rebound tenderness most likely indicating bowel perforation.

Most of the patients failed to correlate foreign body consumption to their medical signs and symptoms, which is expected considering that the median duration from ingestion to the clinical presentation of perforation was 10.4 days, which is compatible with our patient's presentation time [15,16]. Some cases presented months or years after ingesting bone fragments [5,16,17].

Foreign body penetration is a difficult medical situation that is associated with an elevated risk of mortality and morbidity due to late recognition and treatment of the complication [8,13].

Due to the wide range of perforation locations, the common lack of foreign body ingestion recognition, and multiple clinical signs of perforation, making a diagnosis is frequently challenging [14]. Preoperatively, the diagnosis can be achieved only in 23 % of cases [14].

According to reported cases of foreign body perforations, 83 % of these were in the ileocecum, followed by rectosigmoid [10,13,14,18]. These perforation sites are usually associated with peritonitis and abscess development [14,17]. Rarely, fishbone bowel perforation is complicated by the formation of liver abscesses as experienced in our case [14].

A minority of cases of swallowed foreign substances necessitate surgical intervention, while the rest pass through the GI tract without any complications within a week [1,4,13]. Certain foreign bodies have the potential to be lodged, leading to fatal consequences. These involve septicemia, abscess formation, intestinal obstruction, enteric fistulation, and peritonitis [17,18].

The intestine has a protective mechanism that is activated if the mucosa of the intestine gets punctured by sharp-edged substances, causing localized ischemic areas with significant central convexity forms [13]. At the contact site, intestinal wall expansion occurs to facilitate the access of the ingested item [13]. Moreover, in the case of ingesting a lengthy and pointed object the intestinal wall relaxes, and the flow of its contents causes the tip to proceed and the pointy end to tail [13].

Fish bones generate pathological alterations that are unique to their sharp point, which grabs the mucous membrane of the wall of the gastrointestinal tract and leads to a necrotic gut, forming a mucosal band around the fish bone that attaches it to the tissue [13]. Also, the perforated site is concealed by omentum, fibrin, and surrounding bowel loops [10,13].

The main risk factor that is related to bowel perforation caused by fishbone ingestion is denture usage [14,18]. A study demonstrated that dentures are responsible for 80 % of unintentional foreign body consumption [10]. Other precipitating factors include rapid consuming food, extreme age, and cognitive impairment, resulting in unintentional foreign body consumption [14,15,19]. Our patient was mentally stable and failed to recall fish ingestion.

Preoperative diagnosis is challenging, mimicking several abdominal pathologies [4,6,15]. In contrast to other foreign entities, fish bones are difficult to be recognized on plain abdominal radiographs as demonstrated in our case [14]. A study reported the detection of metallic materials by plain radiography [18]. According to Negan et al., the specificity of the plain radiograph in fishbone detection is 91 %, depending on the type of fish [2]. The fishbone was not detected plain abdominal X-ray that was performed for the presented case.

Even though the role of sonography in the identification and detection of fishbone was mentioned in several studies, its usage is often unreliable [14,17]. On ultrasound, foreign substances showed as echogenic spots or streaks can be detected anteriorly below the abdominal wall while the patient is in a supine position [17]. To establish a diagnosis in patients with acute abdominal, a CT scan had a vital role due to its high sensitivity, 86 %, in diagnosing perforated intestinal walls [10,14]. It had the ability to identify calcified and non-calcified foreign substances [10].

The radiological signs of foreign body perforation on CT scan include pneumoperitoneum, curved foreign substance, fatty strands, intraperitoneal effusion, regional inflammatory alterations, and formation of abscess [14,17]. The area around the puncture site frequently has bubbles of air [10,18]. Due to the absence of air bubbles in our case, spontaneous healing process was suspected.

In the current case, a CT scan of the abdominopelvic region showed multiple abscesses that indirectly indicated perforation besides other radiological findings mentioned previously in the case presentation section.

Despite the fact that the medical imaging field is advancing, numerous diagnoses of acute abdominal symptoms can be achieved intraoperatively through surgical procedures such as laparoscopy or open laparotomy [14,15,20]. The gold standard of treatment is surgical techniques to remove foreign objects [10,12,18].

A study reported the role of a patient's early presentation can facilitate an efficient endoscopic resolution of such cases [15]. In the event that an abscess forms and there is a severe inflammatory reaction, it could be challenging to operate during the acute phase. In these situations, conservative therapy may be utilized, postponing surgery until after intravenous antibiotics have been administered [10]. Before surgery, imaging should be performed immediately if the surgical treatment is delayed since the fishbone or other foreign substance may relocate from the original location, affecting surrounding organs negatively [10].

Our case was managed operatively by diagnostic laparoscopy that was converted into exploratory laparotomy due to the presence of multiple abscesses. The patient underwent emergent surgery for intraabdominal abscess drainage with foreign body removal. The intraoperative findings were prescribed in the case presentation section.

## 4 Conclusion

Despite the rarity of foreign body ingestion complications, like perforation, it is related to high morbidity and mortality rates. The bowel perforation and its consequences, following ingesting the foreign substances, are uncommon life-threatening situations. Imaging modalities aid in the demonstration of characteristics and localization of intra-abdominal signs of perforation. To manage foreign body complications, especially perforation, a surgical approach either by laparoscopy or exploratory laparotomy is a viable treatment option. Our case report emphasizes the diagnostic and surgical challenges of foreign materials perforation.

## Funding

No funding or grant support.

## Ethical approval

Not applicable.

## Consent

Written informed consent was obtained from the patient to publish this case report and accompanying images. On request, a copy of the written consent is available for review by the Editor-in-Chief of this journal.

## Research registration

Not applicable.

## Provenance and peer review

Not commissioned, externally peer reviewed.

## CRediT authorship contribution statement

Athary Saleem: paper writing, editing, picture editing, manuscript drafting.

Saqer Alenezi: literature review, paper writing, and editing.

Seddeqah Abdulbaqi: Assist in surgery and editing.

Anas Saud: picture editing.

Nimer Al-Shadidi: performed surgery, paper editing, and supervision.

## Guarantor

Athary Saleem, D.Pharma., B.Med.Sc., M.D., General surgery department, Al-Adan Hospital, Kuwait.

Email: athary.saleem@outlook.com

## Declaration of competing interest

There are no conflicts of interest to declare by all the authors.
